# Spanish Emotion Recognition Method Based on Cross-Cultural Perspective

**DOI:** 10.3389/fpsyg.2022.849083

**Published:** 2022-05-31

**Authors:** Lin Liang, Shasha Wang

**Affiliations:** ^1^College of Foreign Languages and Cultures, Xiamen University, Xiamen, China; ^2^Translation and International Studies Department, Universidad Autónoma de Barcelona, Bellaterra, Spain

**Keywords:** emotion recognition, text processing, natural language processing, BiLSTM, Spanish, cross-cultural

## Abstract

Linguistic communication is an important part of the cross-cultural perspective, and linguistic textual emotion recognition is a key massage in interpersonal communication. Spanish is the second largest language system in the world. The purpose of this paper is to identify the emotional features in Spanish texts. The improved BiLSTM framework is proposed. We select three widely used Spanish dictionaries as the datasets for our experiments, and then we finally obtain text sentiment classification results through text preprocessing, text emotion feature extraction, text topic detection, and emotion classification. We inserted the attention mechanism in the improved BiLSTM framework. It enables the shared feature encoder to obtain weighted representation results in the extraction of emotion features, which enhances the generalization ability of the model for text emotion feature recognition. Experimental results demonstrate that our approach performs better for specialized Spanish dictionary datasets. In terms of emotion recognition accuracy, the average value is as high as 76.21%. The overall performance outperforms current comparable machine learning methods and convolutional neural network methods.

## Introduction

Economic globalization has become a part of our daily life, and linguistic communication is an important part of the cross-cultural perspective. Proficiency in the semantic system of Spanish is a prerequisite for the rapid establishment of cross-cultural communication. It is a necessary skill for cross-cultural personnel involved in diplomacy, foreign trade, and media communications. While language learning is important, the emotions contained in language are the cultural manifestation of a nation. Science and technology have advanced rapidly and Spanish translation systems have become highly sophisticated. However, there are still deficiencies in dealing with the emotions that language contains. Emotion is ubiquitous in our human society and it can be a good way to explain our moods, feelings, and experiences. This is a major characteristic of our human race. For people all over the world, there are commonalities in the expression of emotions, except for the use of facial expressions and body language. Textual language is like a big mountain that separates people’s emotional expressions in different language systems. It is often manifested itself as a language barrier that affects cross-cultural emotional perception and expression from a cross-cultural perspective. Communication between different languages exists not only in the literal sense, but also in the context, and more importantly, in the emotions implied by the text. By grasping the emotional baseline of a text, we can avoid various problems in cross-linguistic communication. There are three ways of dialogue between people, the first way is locutionary, which refers to the actual effect produced between the dialogue; the second way is illocutionary, which refers to the meaning beyond the words between the words; the third way is perlocutionary, which refers to the effect produced after the dialogue is completed. In fact, text emotion recognition has the same steps, the first step is to analyze the literal meaning, the second step is to analyze the extra-verbal meaning, and finally to extract the emotional baseline of the text.

In recent years, researchers have used neural network algorithms to automatically detect large amounts of texts and perform emotional feature recognition. And a large number of industries with application potential have emerged (Strapparava, 2016). It is thus clear that emotion feature computing technology has become the key to emotion recognition intelligent systems. Human emotions can be expressed through facial expressions, body movements, intonation, and speech systems. If computers want to capture and analyze these emotional characteristics, they have to use sensors to acquire the above characteristics and convert them into computer language. Emotion recognition intelligence systems aim to enable computers to automatically calculate emotional characteristics and predict specific human emotional states and behaviors through factors such as speech, video, text, and muscle signals. These predictions are transmitted as input to the secondary system, which analyzes and reacts to these sentiments (Calvo and D’Mello, 2010). Due to the rapid development of the internet, various social media applications have emerged, and people prefer to share their daily feelings and emotions on the internet. Therefore, the text will gradually become an important carrier for people’s emotional attachment. The text data on social media will become an important database for text emotion recognition (Hasan et al., 2019).

The approach of natural language processing is gradually changing. In the past, language processing methods simply connected and tracked the words of the text, and inferred a representation of semantic features from the context. Current language processing approaches perform semantic segmentation based on context and then capture the segmented features from the word level (McCann et al., 2017; Howard and Ruder, 2018). In the form of word expression, each word is expressed as embedded and each embedding form contains contextual semantic segmentation information. The complex features of lexemes are obtained by splitting words and finally modeling based on the sequence of semantic features of lexemes, e.g., a word with multiple meanings. In the current context processing system, Google launched Bidirectional Encoder Representation from Transformers (BERT) (Devlin et al., 2018), which has demonstrated excellent language processing capabilities in a bidirectional context. BERT is a unique neural network using Transformer Encoders (Vaswani et al., 2017). BERT abandons the traditional one-way language model shallow collocation approach and adopts the Masked Language Model (MLM) bi-directional collocation approach, which can efficiently compute and acquire features in bi-directional words natural language. However, BERT requires a certain degree of coherence between sentences and contexts. Because the BERT network has a bidirectional context learning mode, if the contextual information is not coherent, the learned features will be biased and the prediction values will be affected. BERT can currently perform language processing tasks not only in English but can also be extended for multilingual versions. It has a certain foundation for Spanish processing, but when faced with Spanish language text processing tasks, it cannot achieve the language processing effects of English (Pires et al., 2019). To address this issue, more and more researchers have begun to study the multilingual system of BERT, and the processing of the BERT framework in Spanish will be greatly improved in the future (Martin et al., 2019; Virtanen et al., 2019).

Considering that a complete emotion recognition system involves the fusion of expression, voice, and text, the research cost is huge. Therefore, this paper focuses on the emotion recognition of Spanish text. First, we use text pre-processing to convert Spanish text into a suitable tensor; then we complete text emotion feature extraction by the improved BiLSTM framework; then we input all features to the topic detection layer to complete sentence clustering and topic labeling; then we input the emotion classifier to classify the text emotion according to the weights, and finally, we get the result of the emotion classification of the Spanish text.

The remaining sections of this article are organized as follows. Section Related Work discusses the work related to language emotion recognition. Section Materials and Methods describes in detail the relevant principles and implementation process of the Spanish text emotion recognition method. Section Experiments reports the experimental data set, evaluation indicators, and analysis of experimental results. Finally, Section Conclusion summarizes our research and reveals some further research works.

## Related Work

**FIGURE 1:**
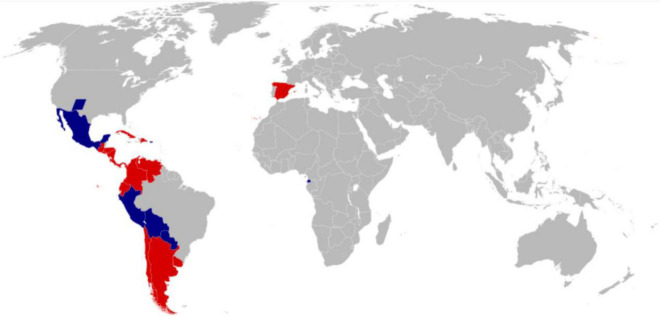
Spanish-speaking regions of the world.

Nowadays, although English is an international language. However, there are about 437 million people in the world who speak Spanish as their mother tongue. Spanish is the second most spoken language system in the world, after Chinese. The Spanish-speaking regions of the world are shown in Figure 1. At the same time, Spanish is one of the six working languages of the United Nations. Therefore, it is of extraordinary significance to understand the emotional messages contained in Spanish texts. If one wants to fully understand the emotions in Spanish texts, one needs to start with the disciplines of Spanish sociology, Spanish linguistics, Spanish history, Spanish philosophy, Spanish ethics, and Spanish psychology. The emotion theory method proposed by Ekman is the basic theory of most current emotion recognition research (Yadollahi et al., 2017). In the research of emotion recognition, a series of models are gradually extended and dedicated to the study of human emotion decomposition. Anger, disgust, surprise, sadness, happiness, and fear are the main recognition baselines for all emotion recognition research. From the perspective of the development of human history, the above six emotions are the basic human emotions, and their expressions are relatively straightforward, so they are easy to express in the work of language text recognition.

In the research of Spanish emotion recognition, feature mining is one of the joint steps. Molina-González proposed a classification system, which is mainly used for the polarity classification of Spanish-specific terms and vocabulary (Molina-González et al., 2015); Martínez-Cámara proposes a classification method based on a machine learning approach to classify social media texts with polarities. Experimental results demonstrate the effectiveness of the method (Martinez-Camara et al., 2015). However, as a small language, Spanish has little research on language and text emotion recognition. Most of the emotion recognition studies for Spanish are based on the SEL dictionary, which is a Spanish text emotion classification dictionary established specifically for Spanish. Due to the limitations of the Spanish emotion recognition dictionary, many researchers have tried to transfer the learning of emotion recognition models from English to Spanish. Redondo has improved on the current English emotion recognition framework ANEW for emotion recognition in Spanish, and the improved method has higher recognition accuracy (Redondo et al., 2007). Plaza-del-Arco discovered the similarities between the NRC strength dictionary (Mohammad, 2017) and Spanish and optimized the dictionary to adapt to the Spanish application (Plaza-del-Arco et al., 2018). In the literature del Arco et al. (2018), the author optimized the WordNet-Affect method for classifying emotion recognition tasks for Spanish, set the corresponding features at each difficulty level, and achieved excellent results in the final difficulty evaluation of emotion recognition.

Currently, the most common methods used in research on language and text emotion recognition are deep learning and machine learning. Commonly used deep learning methods such as long and short-term memory (LSTM) (Hochreiter and Schmidhuber, 1997) and BiLSTN (Schuster and Paliwal, 1997). It has been widely used in end-to-end text emotion classification systems, and it is very effective in encoding text emotion feature sequences. Deep learning methods tend to maintain relatively fast detection speed and detection accuracy. Machine learning methods have higher requirements for designing neural networks. Firstly, the text emotion feature dictionary needs to be classified and then manually labeled with emotion tags. The process of feature training is tedious, but the results of text emotion feature recognition are still good. The method based on the text emotion dictionary has relatively high requirements for the construction of the data set, and it is more labor-intensive. The advantage of this method lies in unsupervised training. For a text database that has been subjected to emotional standards, it can be directly used as the input of this method. The blended learning approach focuses more on the annotation of emotion features of linguistic texts and requires a higher level for the researcher. The researcher must have not only technical skills but also the ability to fully understand the language and culture. The method combines speech, image, and text factors for integrated emotion detection, which can guarantee the accuracy of emotion recognition results to a great extent and is suitable for the overall system application of emotion detection. The main problem of the keyword-based method is the degree of matching between emotion features. At present, the performance evaluation is not as good as the previously mentioned method, so fewer researchers will use this method. Generally speaking, this method is only used in some specific word emotion recognition situations.

Deep learning methods have performed well in research on text emotion classification and are preferred by most researchers. At present, the application of deep learning methods to text emotion classification research is based on the construction of a dataset, which in this case is a linguistic text sentiment dictionary database. In the research of constructing a data set, Rao et al. (2014) presented a neural network approach supplemented with a pruning strategy to automate the construction of textual dictionary databases, and to automatically extract emotion features of dictionary databases at the word level and to automatically encode them. Strapparava (Strapparava and Mihalcea, 2008) focused on the text emotion system for news headlines and constructed a handwritten text emotion lexicon, which achieved good results in fine-grained data evaluation. Neviarouskaya (Neviarouskaya et al., 2007) focuses on text emotion systems in virtual social environments. To achieve text emotion recognition and visualization, rule-based deep learning methods have been properly applied and studied. Banhakawi (Bandhakavi et al., 2017) combined special terms in text data and manually annotated emotion text to extract emotion features of text from neural networks and used the MATCH algorithm for matching to solve the problem of missing emotion features. In the global semantic evaluation contest SemEval, most researchers who study text emotion recognition use shared data sets. Because sharing data sets can significantly save the development time of text emotion recognition algorithms. Moreover, in studies where text datasets are shared, each text word contains manually annotated *a priori* information, so the evaluation results of all the proposed methods are highly informative. In the WASSA2017 conference, some researchers have a great advantage in mining the emotional feature of the textual emotional lexicon (Mohammad and Bravo-Marquez, 2017). However, although great results have been achieved in current research on English text emotion recognition, such as WNA (Strapparava and Valitutti, 2004) and LIWC (Boot et al., 2017). Yet, few models can be successfully transferred to Spanish text emotion recognition.

Zahiri proposed a two-stream LSTM method to obtain the deep connection of text emotional features (Zahiri and Choi, 2018). He accomplished lexeme classification by migration learning and integrated it with the BERT algorithm. Finally, excellent recognition accuracy was obtained in the text emotion dictionary. In the literature Jabreel and Moreno (2019), to transform the text emotion classification problem into a binary classification problem, the authors adopt a deep neural network approach to encode the text sequences and embed an attention mechanism unit and a batch normalization unit. Finally, the classification task is completed by ranking the corresponding emotion features of the matched texts. The experimental results demonstrate a correct classification rate of 96.3%. Chatterjee (Chatterjee et al., 2019) focuses on the study of emotion detection in English conversations. To improve the accuracy of emotion detection, he uses user conversations as the input of the model and divides the input into two sections. Each segment independently corresponds to a different LSTM layer, and the features acquired by each LSTM are different. The first layer of the LSTM sequentially encodes the semantic features to capture the semantic features of the dialogues. The second layer of LSTM traverses the sequence of emotion features to match the emotion word features in the dialogue. Finally, the two outputs are fused and matched, and then the conversation emotion detection results are output.

## Materials and Methods

### Pipeline Overview

**FIGURE 2:**
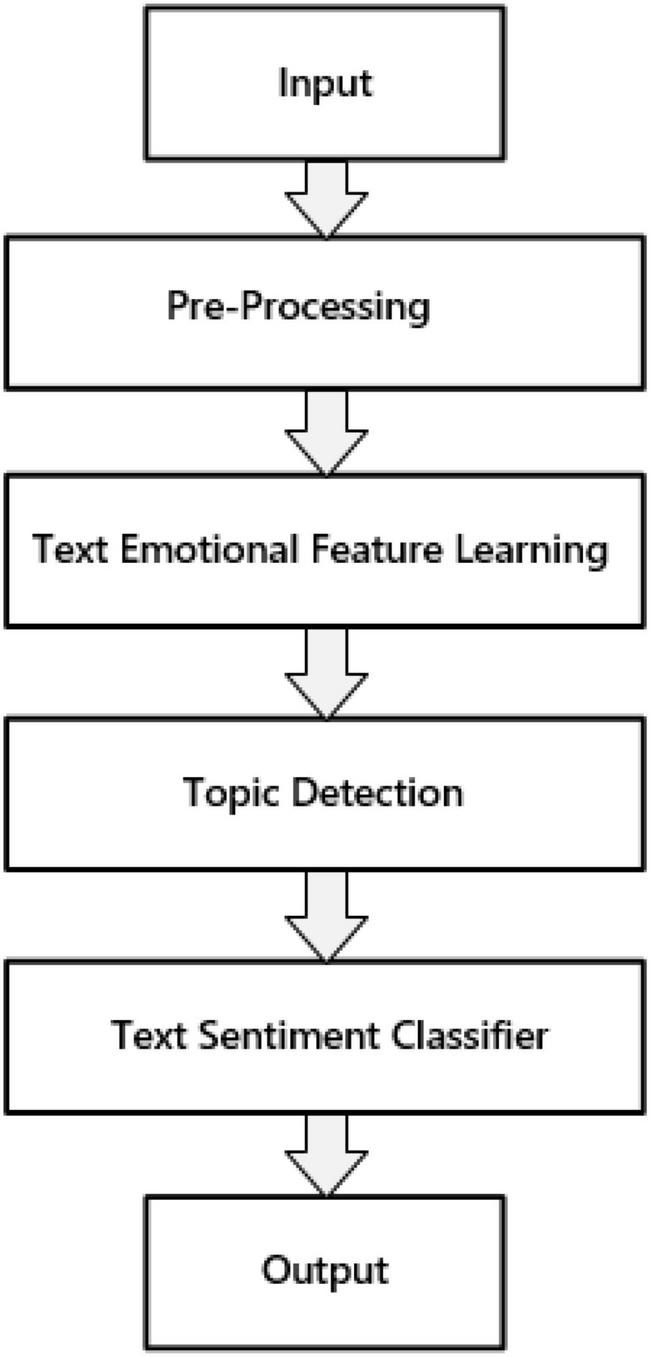
Spanish text emotion recognition process.

We propose an improved BiLSTM framework to recognize the emotions of Spanish text. The overall implementation process of emotion recognition is mainly divided into the following four stages: (1) pre-processing; (2) text emotional feature learning framework; (3) topic detection; (4) text emotion classifier. The overall process is shown in Figure 2.

### Pre-processing

**FIGURE 3:**
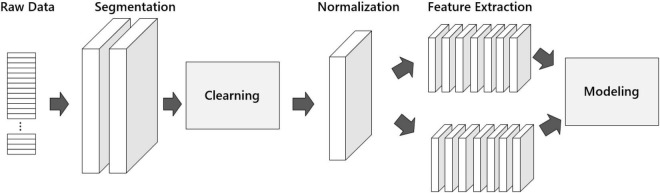
Pre-processing process.

Text preprocessing is a key step in natural language processing. There are many differences between the characteristics of Spanish corpus and English corpus. For example, Spanish can only use the JIEBA library for word segmentation, while English word segmentation only requires spaces. Due to the characteristics of the Spanish corpus, a special preprocessing was performed on the Spanish dictionary dataset. The preprocessing is shown in Figure 3.

In the pre-processing program. First, we input raw data into the word segmentation operation module. The model’s accuracy can be improved by using proper word segmentation, and it can also assist the algorithm to build the correct semantic emotional model. Therefore, the word segmentation operation is a key step. Next, it enters the Cleaning module to eliminate useless tags, special symbols, and deactivated words in the dataset to reduce the probability of text emotion misidentification. Then it enters the Normalization module to ensure the good convergence of the network. And it is input to the feature extraction module to complete the emotion feature extraction of lexemes. N-gram language model and Word2vec distributed model are commonly used for feature processing units. Finally, we perform modeling. We chose a similarity algorithm and a classification algorithm in the modeling. Through text pre-processing, the Spanish data set is converted into a tensor, which provides a reference for the hyperparameter selection of the later model, and improves the overall evaluation index of the model.

### Text Emotion Feature Learning Framework

**FIGURE 4:**
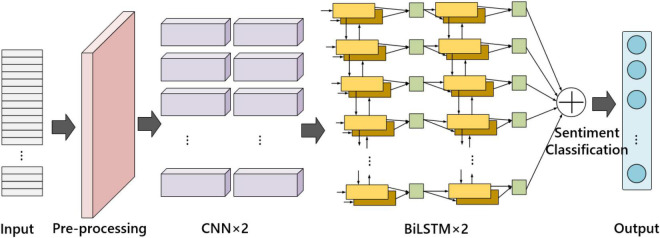
Improved bidirectional long short-term memory (BiLSTM) framework.

In the work of text emotion feature learning, we proposed the contextual bidirectional long short-term memory attention network (we call it improved BiLSTM). For Spanish text emotion recognition, the network is organized into three layers: the input layer, the language-related emotion feature encoder layer, and the text emotion classification layer. The model structure is shown in Figure 4.

Among them, the language-related emotional feature encoder is made up of two layers of neural networks, the first layer is a two-layer convolutional neural network (CNNs), and the second layer is a two-layer bidirectional long short-term memory (BiLSTM). The objective is to learn the spatial distribution of text emotional features from the input sequence features.

In the improved BiLSTM model. To accelerate the convergence of the network and prevent overfitting, we connect a Batch Normalization unit after each convolutional layer; then connect a ReLU activation layer to reduce the computation; and finally, connect a maximum pooling layer to reduce the number of parameters and remove redundant information. The parameter configuration of each convolutional layer is shown in Table 1.

**TABLE 1:** Architecture of improved bidirectional long short-term memory (BiLSTM) layer.

Type	Output dimension	Convolution kernel	Stride	Padding
Convolution	26 × 751 × 128	7 × 7	1 × 1	SAME
Batch normalization	26 × 751 × 128			
Non-linear activation	26 × 751 × 128			
Max pooling	13 × 370 × 128	2 × 2	2 × 2	SAME
Convolution	1 × 370 × 128	13 × 7	1 × 1	VALID
Batch normalization	1 × 370 × 128			
Non-linear activation	1 × 370 × 128			
Max pooling	1 × 370 × 128	1 × 5	1 × 5	SAME

Assuming that the features in the input convolutional layer and finally output high-level semantic features are 1 × 74 × 128 dimensions, and then reshape them into 74 × 128-dimensional features, which are used in the BiLSTM network followed by the feature sequence M, as shown in the following Equation (1).


(1) $$M=\left[m_{1},m_{2},\ldots ,m_{t}\right]$$

Where t represents the length of the characteristic sequence M. LSTM network is an optimization of RNN network, the purpose is to solve the dependency problem of sequence. The LSTM unit includes an input gate _i_t__, a forget gate _f_t__, an output gate _o_t__ and a memory unit _c_t__ to update the hidden state _h_t__, as shown below:


(2) $$i_{t}=\sigma \,\left(W_{i}\,x_{t}+V_{i}\,h_{t-1}+b_{i}\right)$$

(3) $$f_{t}=\sigma \,\left(W_{f}\,x_{t}+V_{f}\,h_{t-1}+b_{f}\right)$$

(4) $$o_{t}=\sigma \,\left(W_{o}\,x_{t}+V_{o}\,h_{t-1}+b_{o}\right)$$

(5) $$c_{t}=f_{t}\,⊙c_{t-1}+i_{t}\,⊙\text{tanh}\,\left(W_{c}\,x_{t}+V_{c}\,h_{t-1}+b_{c}\right)$$

(6) $$h_{t}=o_{t}\,⊙\text{tanh}\,\left(c_{t}\right)$$

**FIGURE 5:**
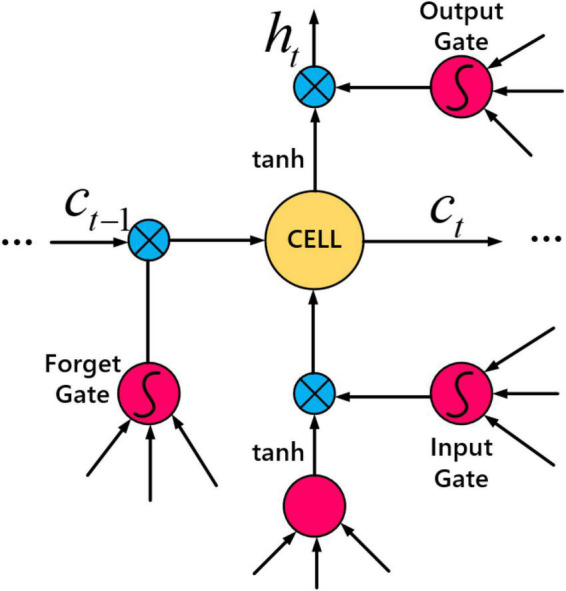
Long and short term memory (LSTM) network.

Where ⊙ is a kind of function which similar to the multiplication operate, *V* represents a matrix related to weight and *b* represents the learning vector. To improve the performance of the model, the morphemes were trained on two LSTMs. The first one is a rightward shifting morpheme starting from the left side, and the next one is a reverse copy of the characters. The outputs of the forward and reverse passes were combined in series before passing to the next layer. Finally, the activation function is used to obtain the prediction results. Figure 5 depicts the LSTM network’s working principle.

In the task of acquiring the feature sequence, to obtain a better prediction model, the contextual relationship needs to be considered at the same time. Two LSTM networks are jointly used, one is used for standard feature sequence learning, and the other is used for reversed feature sequence learning. Finally, the obtained hidden state information is connected to synthesize the final output. In the improved BiLSTM model, the temporal relationship of the input intermediate sequence feature M is modeled by using a two-layer BiLSTM. The hidden vector representation of BiLSTM comes from the forward and reverse LSTM, which are represented by $\vec{h_{t}}$ and $\overset{\leftarrow }{h_{t}}$. The number of hidden layer nodes in each layer of LSTM is 128, and the final hidden vector $h_{t}=\sigma \left(\left[\vec{h_{t},}\overset{\leftarrow }{h_{t}}\right]\right)$ can be obtained by using non-linear transformation at the same time. The obtained output characteristics _h_*t*__ of all time steps are used as the final shared characteristics among different tasks.

### Topic Detection

Topic detection is an intermediate part of text emotion detection, which can map texts to different emotion topics. In the process of theme detection, we combine the analysis methods of different text units. The different text units include the entire document or a simple sentence. In some cases, it is also feasible to encode keywords or terms specifically. In the topic detection model we designed, topic detection is based on the fact that each sentence or paragraph has an individual label, which is determined by the clustering algorithm in preprocessing. In other words, text topic detection represents clustering from paragraph to sentence to phrase. It is further divided into different clusters. The subject objects mapped by each group of clusters are different. After completing sentence clustering, the next task is topic labeling.

We use the hierarchical agglomerative clustering algorithm (HAC) (Schütze et al., 2008) to complete the classification task of Spanish sentences. According to the rules of the HAC algorithm, we need to obtain a text partition to cut into a hierarchical structure. Then, we used different thresholds to define the text hierarchy generated by these cuts and compared the text threshold with the cluster similarity value. According to the comparison result, the similarity is adjusted accordingly. Therefore, the sentence clustering process will be a cyclic difference compensation work. Until the cluster similarity is less than the threshold, the clustering is considered complete. To obtain the threshold value, we defined the standard cut point in the hierarchical structure. According to the literature Shulcloper (2009), two evaluation indicators were proposed for validation. The two evaluation indicators are as follows: (1) The average of the similarity between all morphemes. (2) The average value of the maximum similarity between any morphemes. As shown in the following formula.


$$s\,i\,m\,\left(T_{1},T_{2}\right)$$

$$=\frac{1}{2}(\frac{\sum_{w}\in \left\{T_{1}\right\}\,\left(m\,a\,x\,s\,i\,m\,\left(w,T_{2}\right)*i\,d\,f\,\left(w\right)\right)}{\sum_{w}\in \left\{T_{1}\right\}\,i\,d\,f\,\left(w\right)}$$

(7) $$+\frac{\sum_{w}\in \left\{T_{2}\right\}\,\left(m\,a\,x\,s\,i\,m\,\left(w,T_{1}\right)*i\,d\,f\,\left(w\right)\right)}{\sum_{w}\in \left\{T_{2}\right\}\,i\,d\,f\,\left(w\right)})$$

Where T_*i*_ represents a sentence, and w represents a word contained in the sentence T_*i*_. To compare the sentence similarity of the n-dimensional vector, the cosine similarity is also taken into consideration.

The topic label is a comment on the topic of each sentence cluster, each sentence is clustered according to contextual key phrases, and a simple description of the extracted topic type is called annotation. The area covered by tags is smaller than the topic, so the division of tags under the same topic becomes a challenge. The labels belonging to the clustering methods cannot represent the topics completely and correctly. For example, the sentence centroid method commonly used in sentence clustering operations can only represent sentence features, not topics. Therefore, the later manual correction is particularly important. To figure out this issue, some researchers presented candidate topic models. All topics contained in each cluster are treated similarly as candidate topics, and TF-IDF weighted scores are set in the sentences to measure the relevance between sentences and phrases. Finally, the top three noun phrases are selected as the topics of the sentence clusters for subsequent topic annotation work.

### Text Emotion Classifier

When classifying all the hidden features generated by the BiLSTM model, these features have different responses to different classification tasks. Therefore, the introduction of relevant attention mechanisms allows the classifier to focus on different states in the sequence for different tasks, and to learn a feature representation that is more suitable for the current task. In this way, the accuracy of text emotional feature recognition is improved. In the improved BiLSTM model, the weighted sequence of sentiment features shared by the text sentiment recognition task is represented by $h_{t}=\sigma \left(\left[\vec{h_{t},}\overset{\leftarrow }{h_{t}}\right]\right)$. The representation of this sequence references the sequence model of the attention mechanism.

For the text emotion feature encoder, it is more to extract the shared feature sequence h*_t_* = (h_1_, h_2_, …, h*_t_*), where t represents the length of the sequence. The attention mechanism first calculates the weight $\alpha_{t}^{e}$ corresponding to the feature h*_t_* of each time series t, as expressed by Equation (8).


(8) $$\alpha_{t}^{e}=\frac{e\,x\,p\,\left(f\,\left(h_{t}\right)\right)}{\sum_{t=1}^{t}e\,x\,p\,\left(f\,\left(h_{t}\right)\right)}$$

Where *f*(h) = *W^T^*h, W represents the parameters that can be trained. The output layer _*c^e^*_ of the attention mechanism is the weighted sum of the output sequence, and its weighted expression is as follows.


(9) $$c^{e}=\sum_{t=1}^{t}\alpha_{t}\,h_{t}$$

Finally, the high-level semantic features _*c^e^*_ related to the text emotion are input to the next layer for emotion classification. The classifier of the multilingual emotion recognition task is composed of an emotion-related attention mechanism layer, a fully connected layer (FC), and an output layer. Among them, the FC layer has 128 nodes and uses the non-linear activation function ReLU. To avoid the overfitting issue, a random inactivation layer is added before the output layer, with an inactivation rate of 0.5. The output layer adopts softmax as the activation function. After activation, the emotion category corresponding to the text can be obtained.

## Experiments

### Datasets

**FIGURE 6:**
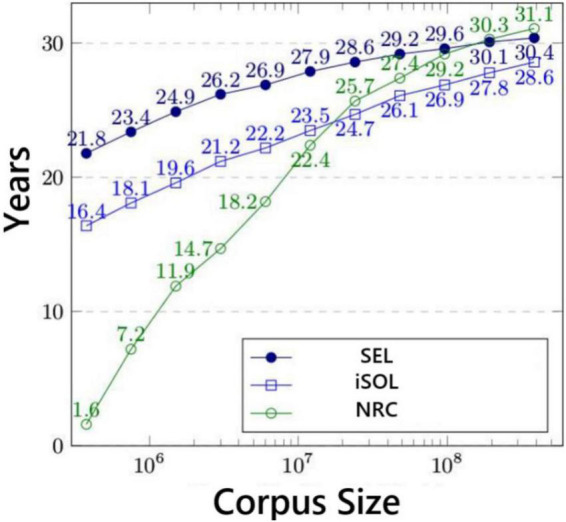
Corpus size distribution of different data sets.

Our experiments mainly involve three data sets, which are NRC Word-Emotion Association Lexicon (EmoLex) (Mohammad and Turney, 2010), Improved Spanish Opinion Lexicon (iSOL) (Molina-González et al., 2013), Spanish Emotion Lexicon (SEL) (Sidorov et al., 2012). The above three corpus data sets have different corpus sizes, and their corpus size distribution is shown in Figure 6.

Among them, NRC is an emotional text data set launched by Google, which is mainly developed for English. However, due to the increasing research on emotions in minor languages, NRC has gradually developed a multilingual version. However, these multi-language versions of text emotion data sets are all generated by Google Translate, and there is a certain gap in accuracy. So, we also chose the iSOL data set, the iSOL data set is a dictionary data set composed of professional Spanish vocabulary and phrases. It was manually proofread by professional Spanish linguists. The application scenario of the iSOL data set is mainly the polarity classification of vocabulary, which is only part of text emotion analysis and cannot cover other parts of text emotion recognition. Therefore, this data set also has certain shortcomings. So, we also merged the third data set SEL. The SEL is also a professional Spanish dictionary data set, all the texts are manually marked by annotators with different thresholds. Each phrase is measured by the standard Probability Factor of Affective (PFA), which corresponds to the emotional performance in different Spanish contexts. In the experimental verification work, we select the above three data sets to evaluate the effect of text emotion recognition, which fully guarantees the comprehensiveness of the data set and the interpretability of the verification results. The number of each emotional phrase in the three data sets is shown in Table 2.

**TABLE 2:** The number of each emotional phrase in Spanish dictionary datasets.

	Datasets		
	NRC	iSOL	SEL
Train	10,217	7,302	1,848
Test	3,024	833	188
Total	13,241	8,135	2,036

### Evaluation Metrics

In the experimental verification process, we choose precision, recall, and F1 score to evaluate the performance of the method. Most classification and sequence labeling problems also adopt the above three evaluation factors. The equation is as follows:


(10) $$F\,1=\frac{2\times p\,r\,e\,c\,i\,s\,i\,o\,n\times r\,e\,c\,a\,l\,l}{p\,r\,e\,c\,i\,s\,i\,o\,n\times r\,e\,c\,a\,l\,l}$$

(11) $$P\,r\,e\,c\,i\,s\,i\,o\,n=\frac{T\,P}{T\,P+F\,P}$$

(12) $$R\,e\,c\,a\,l\,l=\frac{T\,P}{T\,P+F\,N}$$

In the above equation, TP (True Positive) is the number of properly-recognized word segments. FP (False Positive) is the number of misrecognized word segmentation. FN (False Negative) indicates the number of unrecognized word segmentation.

### Experimental Result

We presented a test to verify the efficiency of the revised BiLSTM approach, the recognition tasks were carried out at Valence and Arousal levels respectively, and SVM, RNN, and LSTM methods were selected as the control experimental group. The NRC dataset was used for verification. The experimental results are shown in Table 3.

**TABLE 3:** Text emotion recognition results of different methods at the Valence and Arousal level.

	Valence			Arousal		
	IEMOCAP	RECOLA	Ave	IEMOCAP	RECOLA	Ave
SVM	59.13	56.77	57.95	63.01	58.04	60.03
RNN	65.92	63.32	64.82	68.06	60.51	64.29
LSTM	67.32	65.33	66.26	69.89	59.33	64.61
Ours	71.14	68.65	70.52	74.12	66.13	71.13

Table 3 shows the average UAR rate of 70.52% for our method on the valence recognition task. The average performance of UAR is improved by 12.57% compared to SVM. It can be demonstrated that neural network methods outperform machine learning methods in natural language processing. Among them, the RNN and LSTM methods are inferior to our method for text emotion recognition. It can be seen that the method of fusing CNN and BiLSTM in this paper outperforms the single neural network methods such as RNN and LSTM. In addition, the attention mechanism and multi-task learning structure introduced in this paper enable the model to learn the salient features of different text emotions and mitigate the adverse effects of unaffected factors. Meanwhile, the generalization ability and recognition ability of the model for different emotion features are improved, and the accuracy of emotion recognition is also improved.

In order to represent Valence and Arousal more precisely on the level of emotional characteristics. Extend Valence to emotions of surprise and happiness, and Arousal to emotions of anger and sadness. In the previous step of experimental verification, it has been proved that our method is better than the machine learning method and the same type of neural network method. Therefore, the next step will further verify the emotion recognition effect of this method on the two large Spanish-specific text emotion dictionary data sets, iSOL, and SEL. The experimental results are shown in Table 4.

**TABLE 4:** Text emotion recognition results of our method on the Spanish dictionary datasets.

	NRC			iSOL			SEL		
	*P*	*R*	F1	*P*	*R*	F1	*P*	*R*	F1
Surprise	0.64	0.65	0.68	0.74	0.68	0.69	0.77	0.63	0.69
Happiness	0.66	0.62	0.60	0.71	0.89	0.81	0.70	0.92	0.77
Anger	0.64	0.71	0.69	0.75	0.71	0.72	0.78	0.76	0.77
Sadness	0.68	0.63	0.62	0.75	0.69	0.72	0.78	0.64	0.69

Table 4 shows the emotion recognition effect of our method in the three data sets of NRC, iSOL, and SEL. The experimental results shows that the recognition effect of the NRC data set in the Spanish emotional dictionary is not as good as iSOL and SEL. Because NRC is a data set generated by Google Translate, there is a certain gap between the emotional feature label and the original Spanish emotional feature. Our method is constructed based on the original emotional features of Spanish, so the text emotion recognition effect is better in iSOL and SEL.

To verify the average accuracy of emotion recognition on these three data sets, we have added a new verification experiment. The experimental results are shown in Table 5.

**TABLE 5:** The average recognition accuracy of our method on the Spanish dictionary datasets.

	NRC	iSOL	SEL
Average acc.	70.03	75.53	76.21

The experimental results in Table 5 verify our previous conclusions again. Regarding specialized Spanish text emotion dictionary data sets, such as iSOL and SEL. The average accuracy of our proposed method can reach up to 76.21%, demonstrating the effectiveness of our optimized BiLSTM method in Spanish emotion recognition research.

## Conclusion

In this paper, we propose an improved BiLSTM framework for emotion recognition of Spanish texts. First, we convert the Spanish text into a suitable tensor by text pre-processing; then we complete the text emotion feature extraction by a modified BiLSTM framework; then all features are input to the topic detection layer to complete sentence clustering and topic labeling; then they are input to the emotion classifier to classify text emotion according to the weights, and finally the text emotion classification results are output. Meanwhile, the attention mechanism is inserted in the improved BiLSTM framework. It enables the shared feature encoder to obtain weighted representation results in the process of extracting emotion features, which enhances the generalization ability of the model for text emotion feature recognition. The validation of three major datasets demonstrates the effectiveness of our approach for Spanish text emotion recognition. For the specialized Spanish dictionary dataset, our method performs even better. The average value in emotion recognition accuracy is up to 76.21%. The overall performance outperforms current comparable machine learning methods and convolutional neural network methods.

Considering that our method cannot perform good emotion recognition for long Spanish texts. For the selection of the data set, we will try to choose a special Spanish dictionary data set, so that the results will be more referential. In the next work, we will consider the use of a bidirectional recurrent neural network to enhance our approach to capture the global features of large texts. We will also upgrade our research to the Spanish voice and video level, and finally, build a complete Spanish emotion recognition system to serve the internet industry and the robotics industry.

## Data Availability Statement

The original contributions presented in the study are included in the article/supplementary material, further inquiries can be directed to the corresponding author/s.

## Ethics Statement

The studies involving human participants were reviewed and approved by Xiamen University. Written informed consent to participate in this study was provided by the participants’ legal guardian/next of kin.

## Author Contributions

Both authors designed the whole algorithm and experiments, contributed to the article, and approved the submitted version.

## Conflict of Interest

The authors declare that the research was conducted in the absence of any commercial or financial relationships that could be construed as a potential conflict of interest.

## Publisher’s Note

All claims expressed in this article are solely those of the authors and do not necessarily represent those of their affiliated organizations, or those of the publisher, the editors and the reviewers. Any product that may be evaluated in this article, or claim that may be made by its manufacturer, is not guaranteed or endorsed by the publisher.

## References

[B1] Bandhakavi A. Wiratunga N. Padmanabhan D. Massie S. (2017). Lexicon based feature extraction for emotion text classification. *Pattern Recognit. Lett.* 93 133–142. 10.1371/journal.pone.0194852 29684036 PMC5912726

[B2] Boot P. Zijlstra H. Geenen R. (2017). The Dutch translation of the linguistic inquiry and word count (LIWC) 2007 dictionary. *Dutch J. Appl. Linguist.* 6 65–76. 10.1075/dujal.6.1.04boo

[B3] Calvo R. A. D’Mello S. (2010). Affect detection: an interdisciplinary review of models, methods, and their applications. *IEEE Trans. Affect. Comput.* 1 18–37. 10.3390/s19194079 31547220 PMC6806301

[B4] Chatterjee A. Gupta U. Chinnakotla M. K. Srikanth R. Galley M. Agrawal P. (2019). Understanding emotions in text using deep learning and big data. *Comput. Hum. Behav.* 93 309–317. doi: 10.1016/j.neunet.2019.06.010 doi: 10.1016/j.chb.2018.12.029 31299625

[B5] del Arco F. M. P. Jiménez-Zafra S. M. Martín-Valdivia M. T. Ure^na-L’opez L. A. (2018). “SINAI at SemEval-2018 task 1: emotion recognition in tweets,” in *Proceedings of The 12th International Workshop on Semantic Evaluation*, New Orleans, LA, 128–132.

[B6] Devlin J. Chang M. W. Lee K. Kristina T. (2018). Bert: pre-training of deep bidirectional transformers for language understanding. *arXiv* [Preprint] arXiv:1810.04805,

[B7] Hasan M. Rundensteiner E. Agu E. (2019). Automatic emotion detection in text streams by analyzing Twitter data. *Int. J. Data Sci. Anal.* 7 35–51. 10.1007/s41060-018-0096-z

[B8] Hochreiter S. Schmidhuber J. (1997). Long short-term memory. *Neural comput.* 9 1735–1780. 10.1162/neco.1997.9.8.1735 9377276

[B9] Howard J. Ruder S. (2018). Universal language model fine-tuning for text classification. *arXiv* [Preprint] arXiv:1801.06146, 10.1016/j.jacr.2019.05.007 31173746

[B10] Jabreel M. Moreno A. (2019). A deep learning-based approach for multi-label emotion classification in tweets. *Appl. Sci.* 9:1123. 10.3390/app9061123

[B11] Martin L. Muller B. Suárez P. J. O. Dupont Y. Romary L. de la Clergerie E. V. (2019). Camembert: a tasty french language model. *arXiv* [preprint] arXiv:1911.03894. 10.18653/v1/2020.acl-main.645

[B12] Martinez-Camara E. Martín-Valdivia M. T. Urena-Lopez L. A. Mitkov R. (2015). Polarity classification for Spanish tweets using the COST corpus. *J. Inf. Sci.* 41 263–272. 10.1177/0165551514566564

[B13] McCann B. Bradbury J. Xiong C. Socher R. (2017). Learned in translation: contextualized word vectors. *arXiv* [Preprint] arXiv:1708.00107,

[B14] Mohammad S. M. (2017). Word affect intensities. *arXiv* [preprint] arXiv:1704.08798,

[B15] Mohammad S. M. Bravo-Marquez F. (2017). WASSA-2017 shared task on emotion intensity. *arXiv* [preprint] arXiv:1708.03700, 10.18653/v1/W17-5205

[B16] Mohammad S. Turney P. (2010). “Emotions evoked by common words and phrases: using mechanical turk to create an emotion lexicon,” in *Proceedings of the NAACL HLT 2010 Workshop on Computational Approaches to Analysis and Generation of Emotion in Text*, Los Angeles, CA, 26–34.

[B17] Molina-González M. D. Martínez-Cámara E. Martín-Valdivia M. T. Ureña-López L. A. A. (2015). Spanish semantic orientation approach to domain adaptation for polarity classification. *Inf. Process. Manag.* 51 520–531.

[B18] Molina-González M. D. Martínez-Cámara E. Martín-Valdivia M. T. Perea-Ortega J. M. (2013). Semantic orientation for polarity classification in Spanish reviews. *Expert Syst. Appl.* 40 7250–7257. 10.1016/j.eswa.2013.06.076

[B19] Neviarouskaya A. Prendinger H. Ishizuka M. (2007). “Textual affect sensing for sociable and expressive online communication,” in *Proceedings of the 2nd International Conference on Affective Computing and Intelligent Interaction*, (Berlin: Springer), 218–229. 10.1007/978-3-540-74889-2_20

[B20] Pires T. Schlinger E. Garrette D. (2019). How multilingual is multilingual BERT? *arXiv* [Preprint] arXiv:1906.01502, 10.18653/v1/P19-1493

[B21] Plaza-del-Arco F. M. Molina-González M. D. Jiménez-Zafra S. M. Valdivia M. T. M. (2018). Lexicon adaptation for Spanish emotion mining. *Procesamiento del Lenguaje Nat.* 61 117–124.

[B22] Rao Y. Lei J. Wenyin L. Li Q. Chen M. (2014). Building emotional dictionary for sentiment analysis of online news. *World Wide Web* 17 723–742. 10.1007/s11280-013-0221-9

[B23] Redondo J. Fraga I. Padrón I. Comesaña M. (2007). The Spanish adaptation of ANEW (affective norms for English words). *Behav. Res. Methods* 39 600–605. 10.3758/BF03193031 17958173

[B24] Schuster M. Paliwal K. K. (1997). Bidirectional recurrent neural networks. *IEEE Trans. Signal Process.* 45 2673–2681. 10.1109/78.650093

[B25] Schütze H. Manning C. D. Raghavan P. (2008). *Introduction to Information Retrieval.* Cambridge: Cambridge University Press. 10.1017/CBO9780511809071

[B26] Shulcloper J. R. (2009). *Reconocimiento Lógico Combinatorio De Patrones: Teoría y Aplicaciones. Ph. D. Thesis.* Santa Clara: Universidad Central de Las Villas ‘Marta Abreu’.

[B27] Sidorov G. Miranda-Jiménez S. Viveros-Jiménez F. Gelbukh A. Castro-Sánchez N. A. Velasquez F. (2012). “Empirical study of machine learning based approach for opinion mining in tweets,” in *Proceedings of the Mexican International Conference on Artificial Intelligence*, (Heidelberg: Springer), 1–14. 10.3390/e23070859

[B28] Strapparava C. (2016). “Emotions and NLP: future directions,” in *Proceedings of the 7th Workshop on Computational Approaches to Subjectivity, Sentiment and Social Media Analysis*, San Diego, CA. 10.18653/v1/W16-0430

[B29] Strapparava C. Mihalcea R. (2008). “Learning to identify emotions in text,” in *Proceedings of the 2008 ACM Symposium on Applied Computing*, Fortaleza, 1556–1560. 10.1145/1363686.1364052

[B30] Strapparava C. Valitutti A. (2004). “Wordnet affect: an affective extension of wordnet,” in *Proceedings of the Fourth International Conference on Language Resources and Evaluation Lrec*, Vol. 4 Lisbon, 40.

[B31] Vaswani A. Shazeer N. Parmar N. Uszkoreit J. Jones L. Gomez A. N. (2017). Attention is all you need. *Adv. Neural Inf. Process. Syst.* 30 5998–6008.

[B32] Virtanen A. Kanerva J. Ilo R. Luoma J. Luotolahti J. Salakoski T. (2019). Multilingual is not enough: BERT for Finnish. *arXiv* [Preprint] arXiv:1912.07076,

[B33] Yadollahi A. Shahraki A. G. Zaiane O. R. (2017). Current state of text sentiment analysis from opinion to emotion mining. *ACM Comput. Surv. (CSUR)* 50 1–33. 10.1145/3057270

[B34] Zahiri S. M. Choi J. D. (2018). “Emotion detection on tv show transcripts with sequence-based convolutional neural networks,” in *Proceedings of the Workshops at the 32nd AAAI Conference on Artificial Intelligence*, New Orleans, LA. 10.1155/2021/6694538

